# Prevalence and correlates of severe anxiety in patients with first hospitalization for major depressive disorder combined with dyslipidemia: a large sample cross-sectional study

**DOI:** 10.3389/fpsyt.2023.1289614

**Published:** 2024-01-17

**Authors:** Huimin Yin, Qi Zhang, Yi Li, Jun Ma

**Affiliations:** ^1^Wuhan Mental Health Center, School of Mental Health and Psychological Sciences, Anhui Medical University, Wuhan, China; ^2^Department of Psychiatry, Wuhan Mental Health Center, Wuhan, China; ^3^Department of Psychology, Suzhou Guangji Hospital, The Affiliated Guangji Hospital of Soochow University, Suzhou, China

**Keywords:** dyslipidemia, major depressive disorder, severe anxiety, first hospitalization, prevalence

## Abstract

**Background:**

Anxiety symptoms and dyslipidemia are common co-morbidities in patients with major depressive disorder (MDD), and there are complex pathophysiologic as well as clinical mechanisms underlying the association between the three. In this study, we investigated the prevalence and associated factors of severe anxiety in first-time hospitalized patients with MDD with dyslipidemia.

**Methods:**

We included 708 patients with major depressive disorder with comorbid dyslipidemia and collected their sociodemographic and general clinical data as well as biochemical parameters such as lipids, thyroid function, and blood glucose. We also completed the Hamilton Anxiety Scale (HAMA), Hamilton Depression Scale (HAMD), Positive Symptom Scale (PSS), and Clinical General Impression Scale (CGI) to assess their clinical symptoms.

**Results:**

The prevalence of severe anxiety disorder was 11.02% in patients with MDD with comorbid dyslipidemia. Suicidal history, female gender, body mass index (BMI), HAMD score, PSS score, and FT_4_ level were risk factors for the development of severe anxiety symptoms. Higher HAMD scores, higher PSS scores, and suicidal history were influential factors in exacerbating severe anxiety symptoms.

**Conclusion:**

This study reports and identifies the prevalence of severe anxiety symptoms in first-time hospitalized MDD patients with dyslipidemia, as well as risk factors for anxiety symptoms and factors influencing their severity, and these identified factors may be potentially helpful and informative in preventing and intervening in severe anxiety disorders in this target population.

## 1 Introduction

Major depressive disorder (MDD) is widely recognized as one of the most severe mental health problems (1). Mild depressive episodes are characterized by sadness, lack of pleasure, and feelings of worthlessness, while major depression is characterized by recurrent suicidal thoughts and even suicidal behavior (2, 3). Depression is reported to be the leading cause of mental health-related disease burden and the leading cause of disability globally, affecting approximately 280 million people and causing more than 47 million disability-adjusted life years in 2019 (4). Depression is highly associated with severe anxiety, and a study shows overlapping genetic predisposition between major depressive disorder and anxiety disorders (5). Although they are two separate conditions, depression often occurs in conjunction with severe anxiety, and the relationship between anxiety and depression is complex and may interact with each other (6). This may be because both mood disorders share some similar mechanisms in neurochemistry, cognition, and emotion regulation. From a biological point of view, they both suffer from immune, hormonal, inflammatory, and autonomic system dysregulation (7). In recent studies, anxious depression has emerged as a subtype of MDD and is associated with increased immune dysregulation, more cortical thinning, and cortical limbic dysfunction compared to depression alone (8).

Many studies have shown that poor lipid metabolism and depression are interrelated. An animal study found that high-fat feeding effects induced obesity and hyperlipidemia in mice, which also exhibited depression and anxiety-like behaviors (9). Another study found that the use of statin lipid-lowering drugs produced antidepressant-like effects in rats subjected to chronic mild stress (10). In addition, clinical studies have explored the relationship between lipid levels and depression, with depressed patients being at greater risk for dyslipidemia (11, 12). All of the above studies remarkably suggest that there may be a common pathophysiologic mechanism between dyslipidemia and depressive symptoms. Dyslipidemia may also be associated with alterations in neurotransmitter function and inflammatory responses, factors that play an essential role in the onset and course of anxiety (13). First, dyslipidemia may increase concerns about cardiovascular health, leading to increased anxiety (14). Second, patients with depression often have unhealthy lifestyles, such as lack of exercise and poor diet, which may further affect lipid metabolism and cardiovascular health (15). These poor lifestyle factors may also exacerbate the development of anxiety symptoms.

Several studies have reported an association between abnormal blood lipid levels and anxiety (16, 17). Although some studies have highlighted a possible association between dyslipidemia and anxiety, few studies have investigated the relationship between dyslipidemia and anxiety symptoms in patients with MDD. Risk factors for the development of anxiety symptoms in MDD patients with dyslipidemia remain uncertain. Therefore, it is necessary to use a large clinical sample to determine further the factors influencing the occurrence of anxiety symptoms in the target population. Our objectives were to (1) determine the prevalence and clinical characteristics of anxiety in first-time hospitalized MDD patients with comorbid dyslipidemia. (2) To identify predictive factors significantly associated with anxiety in first-time hospitalized MDD patients with comorbid dyslipidemia.

## 2 Materials and methods

### 2.1 Participants

A total of 708 eligible participants were recruited between 2017 and 2022. Enrolled patients underwent a comprehensive clinical assessment. The inclusion criteria were (1) meeting the diagnostic criteria for MDD in the International Classification of Diseases, 10th edition (ICD-10). (2) According to the 2016 Chinese Guidelines for the Management of Dyslipidemia in Adults, the thresholds for high TC and TG were 5.20 mmol/L and 1.70 mmol/L, respectively, while the thresholds for high LDL-C and low HDL-C were 3.40 mmol/L and 1.00 mmol/L, respectively. Dyslipidemia is considered present when there are single or multiple abnormal lipid levels (18). (3) No history of hospitalization before the inpatient interview on the day of admission. (4) Age between 18 and 60 years, Chinese ethnicity. (5) Hamilton Depression Scale (HAMD) score ≥ 24.

Patients meeting one of the following criteria will be excluded from the study: (1) Pregnant or lactating women. (2) History of drug dependence. (3) History of organic brain disease or endocrine, cardiovascular, and other somatic severe diseases or personality disorders. (4) Inability to cooperate with a psychiatric evaluation due to severe behavioral disorders or other reasons.

### 2.2 Data collection

This study used a cross-sectional design to determine the prevalence of anxiety in first-time hospitalized patients with MDD combined with dyslipidemia. We assessed factors associated with the occurrence of anxiety and compared demographic and general clinical data for two clinical subgroups with and without anxiety. We collected demographic information on each patient, including age, disease duration, gender, marital status and educational background, and history of suicide. We extracted the patient’s body mass index (BMI), waist circumference (WC), and blood pressure level, specifically systolic blood pressure (SBP) and diastolic blood pressure (DBP), from the medical record system. Body mass index (BMI) was calculated by dividing the weight in kilograms by the square of the height in centimeters.

### 2.3 Clinical assessment

We collected scales capable of assessing the clinical symptoms of the patients, namely the Positive Symptom Scale (PSS) (containing items P1 to P7 of the Positive and Negative Symptom Scale), the Clinical General Impression Scale (CGI), and the Hamilton Anxiety Scale (HAMA). We used the HAMA scale to assess anxiety symptoms. Patients with a HAMA score ≥ 25 were defined as the anxiety subgroup (19, 20), otherwise as the no-anxiety subgroup. The PSS scale assessed the severity of psychotic symptoms, with a higher score representing more severe psychotic symptoms. The CGI scale evaluated the efficacy of the treatment and the severity of the condition of the patients, with a higher score representing a poorer treatment effect or a more severe condition.

Before the clinical assessment, two psychiatrists with at least 5 years of clinical experience attended training in using HAMD, HAMA, CGI, and PSS scales.

### 2.4 Laboratory measurements

Our biochemical parameters were extracted from the patient’s inpatient medical record system. All patients were required to fast after 8:00 p.m. the previous day, and blood samples were collected from veins. Blood pressure was measured between 6:00 a.m. and 8:00 a.m. the following day. All blood samples were immediately sent to the hospital laboratory for testing before 11:00 am. Biochemical parameters included total cholesterol (TC), triglycerides (TG), low-density lipoprotein cholesterol (LDL-C), high-density lipoprotein cholesterol (HDL-C), fasting blood glucose (FBG) level, and thyroid function (specifically: thyroid-stimulating hormone (TSH), free tri-iodothyronine (TSH), free tri-iodothyronine (FT_3_), and free tetra-iodothyronine (FT_4_) level).

### 2.5 Data analysis

We removed cases with missing data not included in the statistical analysis. Before running analyses, normality was checked using a Quantile-Quantile Plot. Patient demographics and clinical characteristics were summarized descriptively, *t*-test, *χ*^2^ test, and Mann–Whitney *U* test were used to compare variables between the two groups when appropriate. Binary logistic regression was used to determine risk factors for severe anxiety in patients with MDD. In addition, we constructed ROC curves to assess the predictive value of logistic regression models. Finally, we used multiple linear regression modeling to determine the factors influencing anxiety levels in the target population. We used GraphPad Prism (version 10.1; GraphPad Software, Inc., La Jolla, CA, USA) for graphing and SPSS 26 (SPSS, Inc., Chicago, IL) for statistical analysis.

### 2.6 Ethics statement

Ethics approval and consent to participate.

The ethics committees of the Wuhan Mental Health Center reviewed and approved this study. All subject guardians knew about this study and signed informed consent.

## 3 Results

### 3.1 Differences between clinical subgroups with and without anxiety

The sample consisted of 708 MDD patients with dyslipidemia. Seventy-eight of these patients were defined as having concomitant severe anxiety symptoms. As shown in Table 1, there were significant differences in demographic and general clinical data between the subgroups with and without severe anxiety symptoms. Compared to the subgroup without severe anxiety symptoms, the subgroup with severe anxiety symptoms typically had higher indicators such as score on three scales (HAMD, PSS, and CGI), TSH level, FT_4_ level, FBG level, TC level, LDL-C level, BMI, and blood pressure (SDP and DBP). In the target population, more female than male patients presented with severe anxiety. More patients with severe anxiety had a history of suicidality. However, in terms of disease duration, the subgroup with severe anxiety symptoms had a shorter disease duration.

**Table 1:** Demographic and general clinical data of different clinical subgroups.

Index	Total patients (*n* = 708)	Anxiety		t/χ^2^/z	*p*
		Without (*n* = 630)	With (*n* = 78)		
Age - years	36.09 ± 12.60	35.88 ± 12.59	37.79 ± 12.56	−1.27	0.206
Course of disease - months	10.9 ± 4.56	11.05 ± 4.37	9.79 ± 4.98	2.14	0.035*
HAMD	29.90 ± 2.97	29.41 ± 2.59	33.90 ± 2.79	−14.30	< 0.001*
PSS	7.00 (7.00–7.00)	7.00 (7.00–7.00)	20.00 (12.00–23.00)	−15.31	< 0.001*
CGI	5.92 ± 0.72	5.85 ± 0.61	6.54 ± 0.68	−8.37	< 0.001*
TSH- uIU/mL	3.76 (2.58–4.80)	3.59 (2.54–4.70)	7.38 (4.11–11.50)	−8.49	< 0.001*
FT_3_-mmol/L	4.92 ± 0.67	4.91 ± 0.69	4.96 ± 0.67	−0.67	0.506
FT_4_ - mmol/L	16.77 ± 3.03	16.68 ± 3.00	17.50 ± 3.21	−2.23	0.026*
FBG - mmol/L	5.29 ± 0.65	5.26 ± 0.61	5.56 ± 0.85	−3.01	0.003*
TC - mmol/L	4.98 ± 0.96	4.93 ± 0.94	5.45 ± 0.98	−4.60	< 0.001*
HDL-C -mmol/L	1.30 ± 0.23	1.31 ± 0.23	1.28 ± 0.22	1.17	0.244
LDL-C - mmol/L	2.74 ± 0.80	2.72 ± 0.97	3.00 ± 0.81	−3.00	0.003*
TG - mmol/L	2.45 ± 0.97	2.47 ± 0.97	2.33 ± 0.89	1.16	0.247
BMI - kg/m2	24.20 ± 1.80	24.14 ± 1.77	24.70 ± 1.90	−2.64	0.008*
SBP - mmHg	117.02 ± 11.43	115.96 ± 10.83	125.60 ± 12.53	−6.50	< 0.001*
DBP - mmHg	74.95 ± 7.08	74.35 ± 6.58	79.79 ± 8.98	−5.18	< 0.001*
WC - cm	79.73 ± 8.37	79.65 ± 8.37	80.39 ± 8.36	−0.74	0.451
Gender				5.73	0.017*
Male	230 32.5%	214 34.0%	16 20.5%		
Female	478 67.5%	416 66.0%	62 79.5%		
Education				2.95	0.086
High school and below	504 71.2%	442 70.2%	62 79.5%		
Bachelor and above	204 28.8%	188 29.8%	16 20.5%		
Marital status				0.38	0.537
Unmarried	212 29.9%	191 30.3%	21 26.9%		
Married	496 70.1%	439 69.7%	57 73.1%		
Treatment history				0.64	0.424
NO	256 36.2%	231 36.7%	25 32.1%		
YES	452 63.8%	399 63.3%	53 67.9%		
Suicidal history				186.47	< 0.001*
YES	105 14.8%	53 8.4%	52 66.7%		
NO	603 85.2%	577 91.6%	26 33.3%		

HAMD, Hamilton Depression Scale; PSS, Positive Symptom Subscale; CGI, Clinical Global Impression Scale; TSH, thyroid stimulating hormone; FT_3_, free triiodothyronine; FT_4_, free tetraiodothyronine; FBG, fasting blood glucose; TC, total cholesterol; HDL-C, high-density lipoprotein cholesterol; LDL-C, low-density lipoprotein cholesterol; TG, triglycerides; BMI, body mass index; SBP, systolic blood pressure; DBP, diastolic blood pressure; WC, waist circumference. *p < 0.05.

### 3.2 Determinants of anxiety in MDD patients with dyslipidemia: a binary logic-based model

We constructed a binary logistic regression model (backward: Wald) to explore risk factors for developing severe anxiety symptoms in the target population. We used variables that differed in univariate analyses as independent variables and comorbid severe anxiety symptoms as outcomes. Results showed that Suicidal history (*B* = 1.987, *p* < 0.001, OR = 7.291), Female (*B* = 1.310, *p* = 0.005 OR = 3.704), BMI (*B* = 0.338, *p* = 0.004, OR = 1.402), HAMD score (*B* = 0.243, *p* = 0.003, OR = 1.275), PSS score (*B* = 0.220, *p* < 0.001, OR = 1.246), and FT_4_ (*B* = 0.141, *p* = 0.018, OR = 1.151) were risk factors for severe anxiety symptoms. Table 2 summarizes these results.

**Table 2:** Binary logistic regression analysis of the determinants of anxiety in MDD patients with dyslipidemia.

	Coefficients	Std. error	Wald	*p*	95% CI for EXP (B)		
	B				Exp (B)	Lower	Upper
Constant	−25.871	4.11	31.974				
HAMD	0.243	0.09	8.543	0.003*	1.275	1.083	1.500
PSS	0.220	0.04	36.003	< 0.001*	1.246	1.159	1.338
Gender (Male vs. Female)	1.310	0.07	8.008	0.005*	3.704	1.496	9.157
FT_4_	0.141	0.06	5.564	0.018*	1.151	1.204	1.294
BMI	0.338	0.12	8.161	0.004*	1.402	1.112	1.768
Suicidal history (No vs. Yes)	1.987	0.43	26.267	< 0.001*	7.291	3.425	15.518

HAMD, Hamilton Depression Scale; PSS, Positive Symptom Subscale; FT_4_, free tetraiodothyronine; BMI, body mass index. *p < 0.05.

### 3.3 ROC analysis of factors influencing anxiety in MDD patients with dyslipidemia

**Figure 1:**
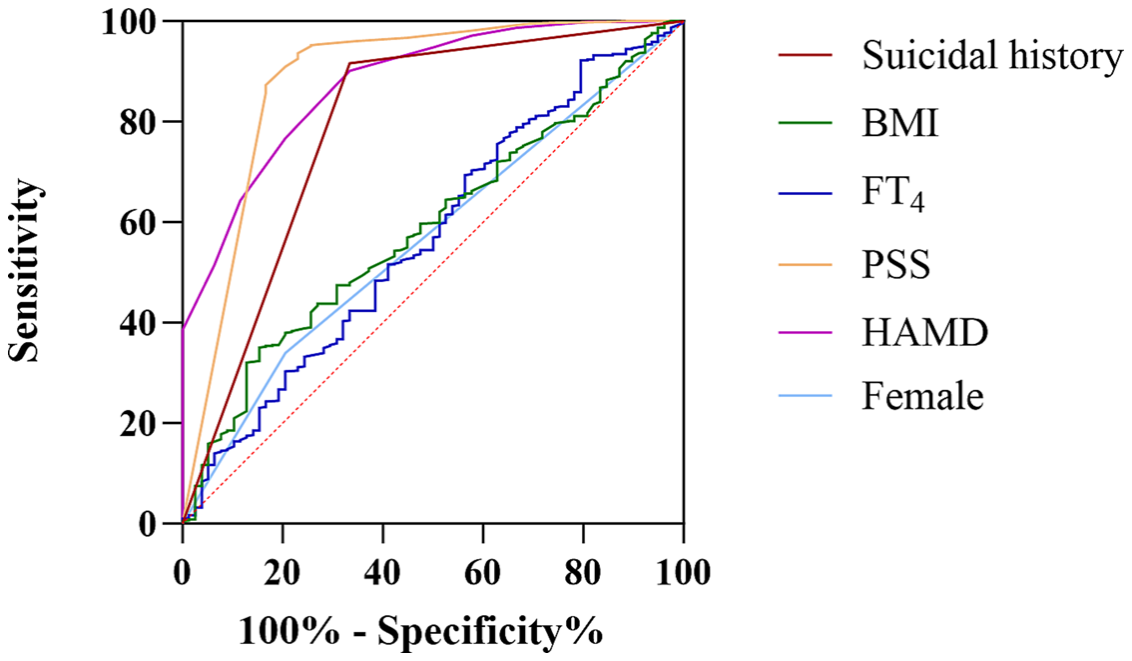
The discriminatory capacity of related factors for distinguishing between MDD patients with comorbid dyslipidemia with and without anxiety symptoms. The area under the curve for suicidal history, BMI, FT_4_, PSS score, HAMD score, and female patients were 0.79, 0.59, 0.51, 0.88, 0.88, and 0.56, respectively.

We performed a ROC analysis of the risk factors identified in the binary logistic regression analysis (Table 2). The areas under the curve for suicidal history, BMI, FT_4_, PSS score, HAMD score, and female patients were 0.79, 0.59, 0.51, 0.88, 0.88, and 0.56, respectively. We concluded that suicidal history (AUC = 0.791, *p* < 0.001, 95% CI: 0.73–0.86), PSS score (AUC = 0.88, *p* < 0.001, 95% CI: 0.83–0.94), and HAMD score (AUC = 0.88, *p* < 0.001, 95% CI: 0.84–0.91) had an excellent discriminatory ability for the presence or absence of severe anxiety in the target population (21), as shown in Figure 1.

### 3.4 Factors affecting levels of severe anxiety in MDD patients with dyslipidemia: a multiple linear regression model

After binary logistic regression analysis (Enter), a total of six factors were included in the regression equation, including HAMD score, PSS score, FT_4_ level, BMI and previous history of suicide, and gender. The correlation coefficient of the regression equation was *R* = 0.734, and the coefficient of determination was *R*^2^ = 0.539. The significance test for the regression equation was *F* = 136.676, *p* < 0.001, indicating that the multiple regression equation was statistically significant. Standardized regression coefficients (*β*) suggested that the shape of the six independent variables on the severity of severe anxiety in the target population, in descending order of magnitude (absolute value), were HAMD score, PSS score, history of suicide, FT_4_ level, gender, and BIM. Two-tailed significance level tests indicated that FT_4_ level, gender, and BIM were not statistically significant (*p* > 0.05), as shown in Table 3.

**Table 3:** Multiple linear regression analysis of factors associated with the occurrence of severe anxiety in MDD patients with dyslipidemia.

	B	Std. error	*β*	*t*	95%CI		*p*	VIF
					Lower	Upper		
Constant	5.004	1.768		2.830	1.532	8.476	0.005	
Gender (Male vs. Female)	−0.167	0.193	−0.022	−0.876	−0.546	0.211	0.386	1.005
Suicidal history (No vs. Yes)	2.217	0.293	0.215	7.256	1.552	2.703	< 0.001*	1.338
HAMD	0.419	0.039	0.354	10.725	0.343	0.496	< 0.001*	1.645
PSS	0.239	0.026	0.320	9.004	0.187	0.291	< 0.001*	1.903
FT_4_	0.037	0.030	0.032	1.229	−0.022	0.095	0.219	1.002
BMI	0.008	0.050	0.050	0.164	−0.091	0.107	0.087	1.009

HAMD, Hamilton Depression Scale; PSS, Positive Symptom Subscale; FT_4_, free tetraiodothyronine; BMI, body mass index. *p < 0.05.

## 4 Discussion

The main findings of the study were as follows: in patients with MDD with comorbid dyslipidemia, (1) the prevalence of anxiety disorders was 11. 02%; (2) there were significant differences between the severe anxiety subgroup and the non-severe anxiety subgroup in terms of duration of the disease, gender, history of suicide, and clinical presentation, such as score of the three scales (HAMD, PSS, and CGI), TSH level, FT_4_ level, FBG level, TC level in the severe anxiety group, LDL-C level, BMI and blood pressure (SDP and DBP) were significantly higher than those of the non-anxiety subgroup. In addition, more females than males suffered from severe anxiety disorders, and the presence of a history of suicide was also higher in patients with severe anxiety disorders; (3) history of suicide, female gender, BMI, HAMD score, PSS score, and FT_4_ level were risk factors for the development of severe anxiety symptoms. (4) HAMD score, PSS score, and a history of suicide were influential factors in the development of severe anxiety symptoms in the target population.

In our study, the prevalence of comorbid anxiety among MDD patients with dyslipidemia was 11.02%. However, to our knowledge, the prevalence of anxiety in MDD patients with comorbid dyslipidemia has rarely been reported. There is a high degree of heterogeneity in the current reports on the prevalence of comorbid anxiety symptoms in MDD patients. For example, one study found that approximately 53.2% of outpatients with major depression also had clinically significant levels of anxiety (22). Among inpatients participating in the third phase of the German algorithm project (GAP3), 46% of patients with depressive episodes had anxiety symptoms (23). In a study on the acute phase cognitive treatment of patients with anxious depression versus non-anxious depression, anxiety symptoms were present in approximately 50.4% of patients with recurrent MDD depression (24). However, in a recent Chinese study of first-episode unmedicated psychotic depression with comorbid anxiety disorders, the prevalence was about 22.8% (25). The heterogeneity of study results may be due to differences in the methods used to assess and define anxiety. For example, the above studies have used HAMD anxiety/panic factor score ≥ seven as an indicator of depression combined with anxiety in their diagnostic assessment (22–24). However, in our study, a HAMA score ≥ 25 was defined as severe anxiety, so it is possible that some patients with anxiety symptoms but not severe enough were excluded from our trial. In addition to different methods of assessing symptoms, our findings may differ due to the inclusion of other populations, ethnicity, and differences in gender composition. In conclusion, the prevalence of severe anxiety in our study population was not high.

We also found that HAMD score, PSS score, body mass index, history of suicide, female gender, and FT_4_ level were risk factors for severe anxiety symptoms. Using ROC curves, we also found that a history of suicide, PSS score, and HAMD score had good predictive ability for the presence of severe anxiety in MDD patients with dyslipidemia. There is a substantial co-morbidity between anxiety and depression, as we believe. As the severity of depression progresses, anxiety-related symptoms usually increase (26). In clinical practice, depression and anxiety disorders often coexist. Studies have shown that up to 50–60% of patients with depression also have anxiety symptoms (27).

Furthermore, previous studies have shown that patients with MDD who have psychotic symptoms are more likely to experience anxiety. A study has shown that patients with MDD with psychotic symptoms have a 14.89-fold increase in the prevalence of severe anxiety (24.28%) compared to patients with MDD without psychotic symptoms (25). And Koyanagi et al. found that anxiety was significantly associated with coexisting psychotic symptoms in patients with depression (28), Gaudiano et al. found a higher prevalence of specific anxiety disorders in patients with major depressive disorder who had psychotic symptoms (29).

Some studies also have found that elevated BMI predicts the long-term development of depression and anxiety symptoms (30, 31). In rodent model studies, rats prone to obesity exhibit higher levels of anxiety-like behavior compared to resistant rats when maintained on a standard diet (32). Although it is widely accepted that obesity is associated with the onset of depression, the exact mechanisms by which these two disease entities interact remain unclear. Gut microbial imbalances, inflammatory responses as emotional disorders, and obesity are now well established (33–35). In addition, one study found that the dorsal striatal terminal dorsal bed nucleus was found to play a crucial role in the reciprocal control of the co-morbidity of obesity and mood disorders. High-fat diet-mediated desensitization reduces *GABAergic* output from *AgRP* neurons to downstream melanocortin four receptors in the dorsal striatal terminal dorsal bed nucleus neurons, leading to severe mood disorders (36).

In general agreement with previous reports, patients with anxiety depression had a higher frequency of major depressive episodes and a higher risk of suicidal ideation and previous suicide attempts compared to patients with non-anxiety depression (23, 37). A review explains the functional characteristics and nature of 5-HT receptor involvement in the regulation of pathological behavior, highlighting the role of 5-HT receptors in behavioral conditions such as suicide, depression, and anxiety (38). In terms of the risk of the disease by gender, it has been suggested that women are more prone to anxiety than men (39). A recent Chinese epidemiologic survey found a statistical difference in the prevalence of severe anxiety between men and women with patient MDD (9.79% vs. 22.12%) (40).

Our study also demonstrated that FT_4_ levels are a risk factor for severe anxiety in MDD patients with dyslipidemia. Regarding the association between FT_4_ and anxiety symptoms, studies have reported that higher FT_4_ in subclinical hyperthyroidism and within the normal range is associated with poorer cognitive outcomes (41). Another animal experiment found that a high level of FT_4_ may induce anxiety and depressive behavior in rats (41, 42). However, there is some heterogeneity in the presence of relevant studies. For example, no significant differences in serum FT_4_ levels were observed between MDD patients with and without anxiety in the study by Zhang et al. (43). The relationship between FT_4_ and anxiety symptoms is unclear.

We finally also identified HAMD score, PSS score, and history of suicide as factors influencing the severity of anxiety symptoms in the target population. Consistent with previous findings, in patients with first-episode unmedicated MDD, Zhang et al. found that psychotic symptoms and suicide attempts significantly predicted MDD with severe anxiety symptoms (44). It has also been found that the PASS score seems to represent a good indicator of anxiety, as it is significantly correlated with more systematic measures of anxiety (45). Our report suggests that the HAMD score is predictive of severe anxiety symptoms, and previous studies have found that anxiety manifests itself at a higher HAMD score and increases with increasing depression severity. Anxiety symptoms cannot be well differentiated at a lower HAMD score (26). However, since our study sample was patients with major depression and all HAMD scores≥25 (19), it supports our results.

Our study has several limitations. First, this cross-sectional preliminary study could not explain the causal relationship between anxiety severity and risk factors in MDD patients, which needs to be confirmed in future prospective cohort studies. Second, due to the many causes of anxiety symptoms, we were only able to include a subset of variables as independent variables and did not analyze the effects of physical condition, social and family support. Thirdly, as this study was conducted on patients hospitalized for the first time, who are usually in the acute phase of the disease, our study cannot be generalized to patients in the stable phase of the disease. Finally, because this was a cross-sectional study, we focused only on studies related to MDD patients with dyslipidemia and did not include MDD patients with normal lipids. Therefore, we cannot know whether these factors affecting anxiety symptoms are specific to patients with dyslipidemic MDD or are also prevalent in patients with normolipidemic MDD. Future studies should aim to control for these confounding factors, and further investigation of the prevalence of anxiety disorders and their influencing factors in patients with dyslipidemic MDD is needed to make our study more meaningful.

In conclusion, the prevalence of severe anxiety symptoms among first-time hospitalized MDD patients with dyslipidemia was 11.02%. We found that HAMD score, PSS score, history of suicide, BMI, female, and FT_4_ were risk factors for severe anxiety symptoms in this patient group. In addition, HAMD score, PSS score, and history of suicide were predictors of the severity of anxiety symptoms in the target group. These identified factors may provide potential biological indicators for clinical intervention and prevention of severe anxiety symptoms in this population.

## Data availability statement

The original contributions presented in the study are included in the article/supplementary material, further inquiries can be directed to the corresponding authors.

## Ethics statement

The studies involving humans were approved by the Ethics Committee of Wuhan Mental Health Center. The studies were conducted in accordance with the local legislation and institutional requirements. The participants provided their written informed consent to participate in this study.

## Author contributions

HY: Writing – original draft. QZ: Formal analysis, Writing – review & Editing. YL: Investigation, Supervision, Writing – review & editing. JM: Conceptualization, Methodology, Software, Investigation, Formal analysis.
